# Stay Fit, Stay Young: Mitochondria in Movement: The Role of Exercise in the New Mitochondrial Paradigm

**DOI:** 10.1155/2019/7058350

**Published:** 2019-06-19

**Authors:** Jesus R. Huertas, Rafael A. Casuso, Pablo Hernansanz Agustín, Sara Cogliati

**Affiliations:** ^1^Institute of Nutrition and Food Technology, Biomedical Research Centre, Department of Physiology, University of Granada, Granada, Spain; ^2^Centro Nacional de Investigaciones Cardiovasculares (CNIC), Madrid, Spain

## Abstract

Skeletal muscles require the proper production and distribution of energy to sustain their work. To ensure this requirement is met, mitochondria form large networks within skeletal muscle cells, and during exercise, they can enhance their functions. In the present review, we discuss recent findings on exercise-induced mitochondrial adaptations. We emphasize the importance of mitochondrial biogenesis, morphological changes, and increases in respiratory supercomplex formation as mechanisms triggered by exercise that may increase the function of skeletal muscles. Finally, we highlight the possible effects of nutraceutical compounds on mitochondrial performance during exercise and outline the use of exercise as a therapeutic tool in noncommunicable disease prevention. The resulting picture shows that the modulation of mitochondrial activity by exercise is not only fundamental for physical performance but also a key point for whole-organism well-being.

## 1. Introduction

Commonly speaking, exercise is a physical activity that works the body at greater intensity than usual movements. To work at higher intensity, the body, particularly the skeletal muscles and the cardiovascular system, requires an extra supply of energy. Mitochondria are the main energy suppliers that coordinate AT production, reactive oxygen species (ROS) production, and calcium signaling, which are fundamental processes for sustaining body activity during exercise. Thus, exercise and mitochondria are in a close relationship and influence each other. This review is focused on this relationship, providing an overview of the most relevant works in the literature. Moreover, since diet can influence mitochondrial activity, we discuss how the use of some nutraceutical products can modulate physical performance. Lastly, we underline the use of exercise as a cost-effective primary intervention to prevent and delay metabolic syndrome and cardiovascular disease. Thus, the exercise-mitochondria interaction is not only an energetic matter but also an important aspect that must be understood to improve the use of exercise for both community members and athletes.

## 2. Mitochondria: The Hub of Energy Production

Mitochondria are defined as the energetic center of cells because they are the site of the oxidative phosphorylation (OXPHOS) system. The OXPHOS system is located in the mitochondrial cristae and is composed of the complexes of the electron transport chain (ETC) and the ATP synthase. The four complexes of the ETC are complex I (NADH-ubiquinone oxidoreductase), complex II (succinate-quinone oxidoreductase), complex III (ubiquinol-cytochrome *bc*_1_ oxidoreductase), and complex IV (cytochrome *c* reductase). Using ubiquinone and cytochrome *c*, these complexes couple electron transport with proton pumping to generate a gradient that is used by ATP synthase to phosphorylate ADP and produce ATP. For decades, it was thought that the mitochondrial respiratory complexes were found as isolated entities [1]. However, it is currently widely accepted that respiratory complexes can be assembled into supramolecular entities called supercomplexes (SCs) [2, 3] whose structures have been described in detail, though not fully characterized, by several important and elegant studies involving cryo-EM [4–6]. SCs have been described in different organizations from yeast to plants and mammals [2, 7–10]. Accordingly, in the *plasticity model*, SCs and complexes can coexist and have dynamic interactions [7] that can be modulated by tissue-specific subunits [11] according to the energetic requirement of the cells [12, 13]. It has been demonstrated that SCs can (i) enhance the efficiency of electron flux by segmentation of the CoQ pool [14], (ii) prevent ROS formation by complex I and complex III as demonstrated by Lenaz and Genova [15] and (iii) improve the stability of the individual respiratory complexes [16–18]. However, many questions remain in the field, particularly if SC assembly is necessary for enhancing mitochondrial respiratory efficiency and what role SCs play in sustaining and determining metabolism. More evidence is required to definitively answer these questions and define the roles of SCs.

The discovery of the first *bona fide* SC assembly factor, supercomplex assembly factor 1 (SCAF1) [12], demonstrated that SCs are genetically regulated structures. SCAF1 is a small protein of 13 kDa that is homologous to the CIV subunit Cox7a2. SCAF1 brings together CIII and IV. Surprisingly, CD1 and SV129 mice have the wild-type protein, while C57BL/6J and Balb/cJ mice suffer a mutation that displaces a critical histidine residue in the matrix-exposed domain that is important for the interaction with CIV. As a result, liver mitochondria from C57BL/6J and Balb/cJ mice do not have III_2_+IV and I+III_2_+IV (either defined as the respirosome). This different organization of the respiratory chain determines higher respiration rates and ATP production in C57BL/6J and Balb/cJ mice when fueled with pyruvate/malate (NADH route) or succinate (FADH_2_) that cannot be increased with the simultaneous fueling of both substrates as in CD1 and SV129 mitochondria. Our proposed model suggests that SCAF1, by defining three different populations of IV (the first linked to I and receiving the electron from the route of NADH, the second associated with III_2_ and dedicated to the route of FADH_2_, and a third that can receive the electrons from both routes), prevents saturation of electron channeling with one substrate, promoting the optimal use of energetic substrates, and suggests the existence of preferential pools of coenzyme Q (CoQ).

However, since the heart and muscle mitochondria from C57BL/6J mice retain a significant amount of respirosome [11], some authors doubt that SCAF1 is required for its formation [19, 20] and for the channeling of electrons through preferential pools of CoQ. From a structural point of view, using extensive proteomic analysis, we demonstrated that the respirosome from BL6 mice incorporates the COX7A2 subunit, which is specific to the monomeric status of complex IV, instead of SCAF1, suggesting that different respirosomes can exist in which CIII and IV undergo different interactions [11]. These results could be in agreement with the most recent cryo-EM structures of Letts et al.'s group, in which a loose and tight form of I+III_2_+IV was described [4]. Regardless, the debate is still open, and a deep analysis of the structures, compositions, and functions of these SCs is urgently needed to understand whether these two structures are indeed different SCs with different functions and metabolic outcomes.

Another important element for the organization of the SCs of the ETC is crista morphology [21]. Cristae are specialized compartments of the mitochondrial inner membrane that are considered to be the platform on which SCs are clustered and assembled. Their remodeling is a tool for modulating mitochondrial metabolism and regulating the assembly and stability of SCs [21, 22]. It has been known that mitochondria continuously modulate their shape in an equilibrium between fusion and fission to maintain their function and eliminate damaged mitochondria during the so-called mitochondrial life cycle [23]. However, increasing evidence has demonstrated that adjusting morphology is a strategy to modulate mitochondrial bioenergetics and cope with the energetic demand of cells [24]. In the presence of excess nutrients, mitochondria divide to uncouple the electron transport chain and reduce ATP production [25], as occurs after treatment with uncouplers. On the other hand, mitochondria elongate in response to sustained deprivation of nutrients and escape from massive autophagy [26] that would ultimately be detrimental for the cell. In both situations, the molecular mechanisms, although not fully identified, involved the family of mitochondrial shaping proteins in which OPA1 processing and DRP1 recruitment control mitochondrial fission, while mitofusin expression and OPA1 cooperate for mitochondrial fusion [27].

Changes in mitochondrial morphology and remodeling of the SCs' structure during exercise will be discussed in detail in the following sections.

## 3. The Energetic Demand of Exercise

Skeletal muscle contractile activity is directly dependent on the supply of adenosine triphosphate (ATP) to three ATPases: myosin, Na+-K+, and sarcoplasmic reticulum Ca2+ [28].

**Figure 1:**
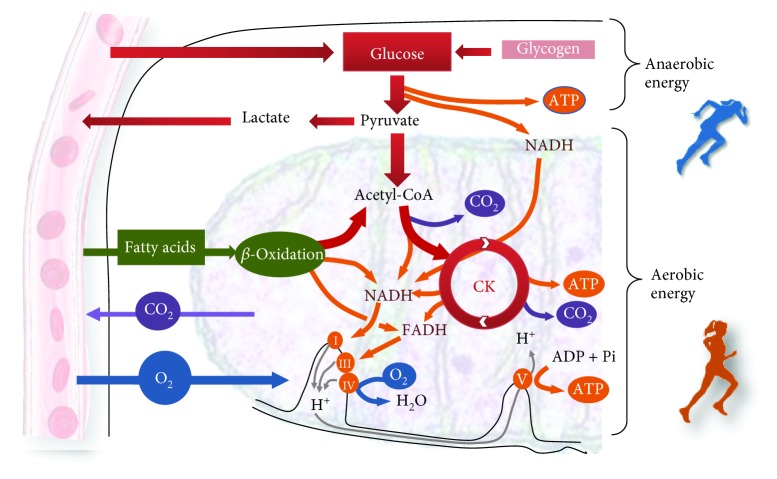
Energy system contribution during exercise. CK: Krebs cycle; I, III, IV: mitochondrial complexes; V: ATP synthase: FADH: reduced flavin adenine dinucleotide; NADH: reduced nicotinamide adenine dinucleotide; ATP: adenosine triphosphate; ADP: adenosine monophosphate.

However, the amount of mammalian intramuscular ATP allows the maintenance of high-intensity exercise for only a few seconds. Therefore, from a bioenergetic point of view, exercise constitutes an energetic challenge within skeletal muscle cells. Skeletal muscle possesses a number of ATP synthetic pathways that permit high ATP turnover even during strenuous exercise. These pathways engage both anaerobic, substrate-level phosphorylation, and aerobic metabolism, which is highly dependent on the oxygen delivered by the cardiovascular system to the skeletal muscle [28] (Figure 1).

**Figure 2:**
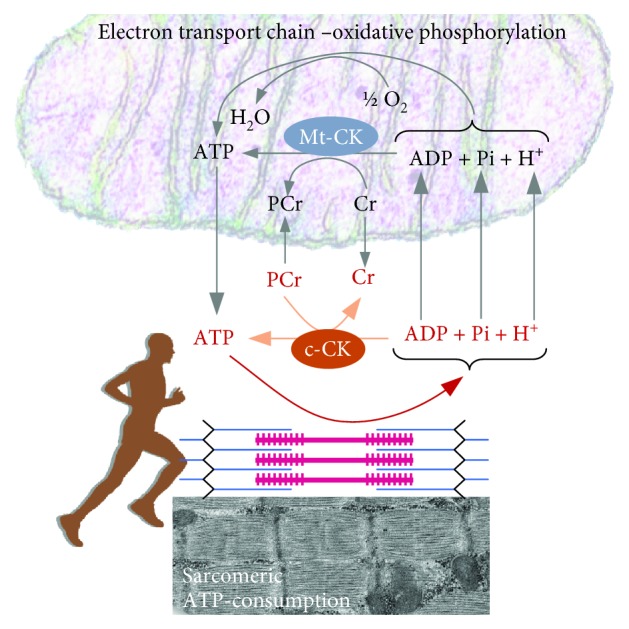
The creatine kinase/phosphocreatine system. Compartment-specific isoenzymes of creatine kinase (CK) are found in mitochondria (Mt-CK) and cytosol (c-CK). PCr: phosphocreatine, Cr: creatine, ATP: adenosine triphosphate, ADP: adenosine monophosphate.

During the first seconds of intense exercise, ATP is produced at the substrate level via creatine kinase (CK), an enzyme mainly present in the cytosol that catalyzes a reversible reaction in which phosphocreatine (PCr) converts adenosine diphosphate (ADP) to ATP, forming creatine (Cr) (Figure 2):

(1) $$\begin{array}{c} \text{ADP}+\text{PCr}↔\text{Cr}+\text{ATP} \end{array}$$

As CK is high in cytosol, small increases in ADP trigger the degradation of creatine phosphate, leading to ATP production.

However, during high-intensity exercise, this system provides ~50% of the ATP during the first 6 s of exercise, while the most predominant substrate for the next 10 s is glycolysis [29]. These events are concomitant due to an increase in sarcomeric (Ca^+^) and inorganic phosphate (Pi), leading to the production of high levels of pyruvate, mainly due to glycogenolysis. These pathways are activated in the cytosol; however, pyruvate can either be metabolized in the cytosol and produce lactate [30] or enter into the mitochondria for oxidation (i.e., anaerobic or aerobic glycolysis, respectively). Indeed, glycolysis by-products activate the main mitochondrial enzyme responsible for carbohydrate oxidation, pyruvate dehydrogenase (PDH) [29]. If exercise intensity continues to increase, the glycolytic efflux also increases, and pyruvate production exceeds the oxidation rate of PDH, leading to lactate generation. Therefore, the degree of oxidative metabolism during exercise is inversely related to the intensity of exercise. For instance, sprint running events lasting <10 s, such as world-class 100 m running, can be sustained using mainly PCr hydrolysis. Events up to ~60 s, such as 400 m running or 100 m swimming, mainly use anaerobic glycolysis, while longer races mainly use aerobic metabolism.

Although anaerobic glycolysis starts as soon as muscle contraction starts, its contribution is mainly from 10-60 s of intense exercise [31]. This process requires the stored glycogen to produce lactic acid and ATP. Thus, the importance of adequate skeletal muscle glycogen levels before intense exercise is highlighted as follows:

(2) $$\begin{array}{c} \text{Glycogen}\to \text{glucose}-1-\text{P}\to \text{lactate}+\text{ATP} \end{array}$$

Exercise beyond 60 s requires oxygen to oxidize glucose or fatty acids to carbon dioxide:

(3) $$\begin{array}{c} \text{Glucose}/\text{fatty acid}+\text{oxygen}\to \text{carbon dioxide}+\text{water}+\text{ATP} \end{array}$$

**Figure 3:**
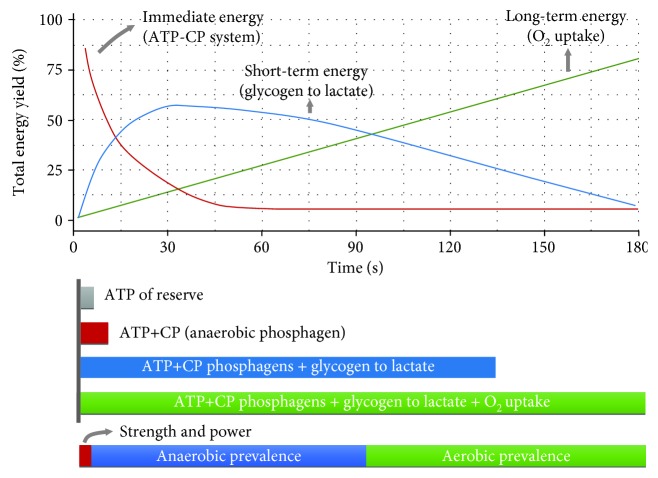
The energy systems that contribute to sport practice. The phosphagen system (ATP-CP) is used in explosive movements (Immediate energy). Anaerobically created energy overlaps with use of the ATP-CP system to provide energy for activities lasting up to around 3 minutes. Aerobic glycolysis provides energy for longer-distance events by breaking down fat and some carbohydrates.

As oxygen is a key factor in the latter metabolic pathway, mitochondria play a predominant role during most exercise conditions. It should also be noted that even if 30 s sprints are repeated over time, after the third repetition, most of the ATP is provided by oxidative mechanisms [29]. Therefore, some of the mitochondrial adaptations related to sprint exercise (see below) can be attributed to the fact that a repeated effect compromises oxidative phosphorylation. Nevertheless, during prolonged submaximal exercise, the oxidative metabolism of carbohydrates and lipids almost fulfils all the cellular ATP needs, but the relative contribution of each substrate (i.e., fatty acids and glucose) is also a matter of exercise intensity. The maximal fat oxidation rates occur at intensities ~60% of VO_2_max [32], but as intensity increases, the glycolytic flux increases due to decrements in plasma fatty acid (FA) delivery to contracting muscles and a decreased capacity for mitochondrial FA uptake and oxidation [33]. It should, however, be noted that PCr hydrolysis, glycolysis, and oxidative phosphorylation work simultaneously during exercise and that the relative contributions of these components vary in response to exercise intensity (Figure 3).

## 4. The Role of Mitochondria in Exercise

Mitochondria are the organelles where oxidation meets phosphorylation to generate ATP for contracting muscles. In fact, since the early studies by John O. Holloszy [34], it has been clear that exercise elicits mitochondrial adaptations [35]. In addition, subsequent studies in rodents demonstrated that the increase in mitochondrial enzyme activity induced by exercise first observed by Holloszy (1967) was due to increases in mitochondrial size and number [36]. These pioneering studies suggested that the maximal oxygen uptake (VO_2_max) and the whole-body aerobic capacity were also limited by skeletal muscle mitochondrial content and not only by cardiorespiratory factors, as previously thought. Accordingly, some studies have linked skeletal muscle mitochondrial content and ATP supply with skeletal muscle performance [37, 38]. This finding suggests that mitochondria may set the limit of maximum O_2_ uptake by skeletal muscle. This assumption makes sense because mitochondria link oxygen delivery to ATP synthesis to meet the energetic needs of the skeletal muscle. Therefore, exercise training is a key factor that can enhance mitochondrial bioenergetics efficiency.

In fact, endurance exercise training can increase the mitochondrial content per gram of tissue as well as the mitochondrial composition (protein-to-lipid ratio), thereby improving aerobic metabolism [39]. In addition, exercise training can improve mitochondrial metabolism by increasing the ATP supply per O_2_ molecule (P/O). In fact, oxidative phosphorylation is a highly malleable biochemical process [40], in which the ETC oxidizes NADH to pump H+ through the inner mitochondrial membrane. Then, the proton motive force is used by the F_1_F_0_-ATP synthase to produce phosphorylation. Another step that can be optimized within trained skeletal muscle mitochondria is H+ leakage through the inner mitochondrial membrane, thus bypassing phosphorylation. Overall, aerobic exercise training improves skeletal muscle O_2_ uptake, which directly affects aerobic exercise performance, thereby increasing fatty acid (FA) oxidation and limiting lactate production at a given exercise intensity. This effect can occur due to a rise in skeletal muscle content and size but also due to improvements in the oxidative phosphorylation system.

It is important to highlight that two different pools of mitochondria exist within the skeletal muscle and the heart. The subsarcolemmal (SS) mitochondria are located close to the sarcolemma, while intermyofibrillar (IMF) mitochondria are located between myofibrils. Notably, SS mitochondria are often in close contact with the peripherally located mitochondria (i.e., functional) myonuclei. Therefore, it has been proposed that the main function of SS mitochondria is to energetically support nuclear and sarcolemmal functions [41]. However, IMF mitochondria show higher biochemical activity and ATP concentrations [42], possibly to efficiently provide ATP for myosin ATPase, thereby sustaining myofibril contraction. With regard to exercise training, a higher trainability of SS than IMF mitochondria has been observed. In fact, the volume density of SS mitochondria is ~0.1-0.2% in untrained subjects, which can increase up to ~3.4% in highly trained subjects [43]. However, the IMF volume density is ~4.5% in untrained subjects, while increases up to ~7.5% have been described [43].

Therefore, it can be inferred that in response to exercise, SS mitochondria increase their volume to a greater extent than do IMF mitochondria. However, the rationale for these divergent responses to exercise between mitochondrial subpopulations is unknown. A possible explanation matches the power-transmitting cables, which suggest that oxygen from capillaries is consumed by SS mitochondria and that the ions can then be pumped to IMF mitochondria [44]. Therefore, the power-transmitting cables can energetically interconnect oxygen-rich areas, such as those close to the sarcolemma, and deeper areas of the muscle fiber. This adaptation has not been directly observed in response to exercise. However, the enlargement of the mitochondrial reticulum reported in response to exercise [45–47] together with the stronger adaptations described within SS mitochondria suggests that trained skeletal muscle may be adapted towards improving the distribution of energy through skeletal muscle cells. Nevertheless, given that little on the SS and IMF response to exercise has been reported, in the present review, we will discuss the general response of the mitochondrial reticulum.

## 5. Exercise Influences Mitochondria, Which in Turn Influence Exercise

**Figure 4:**
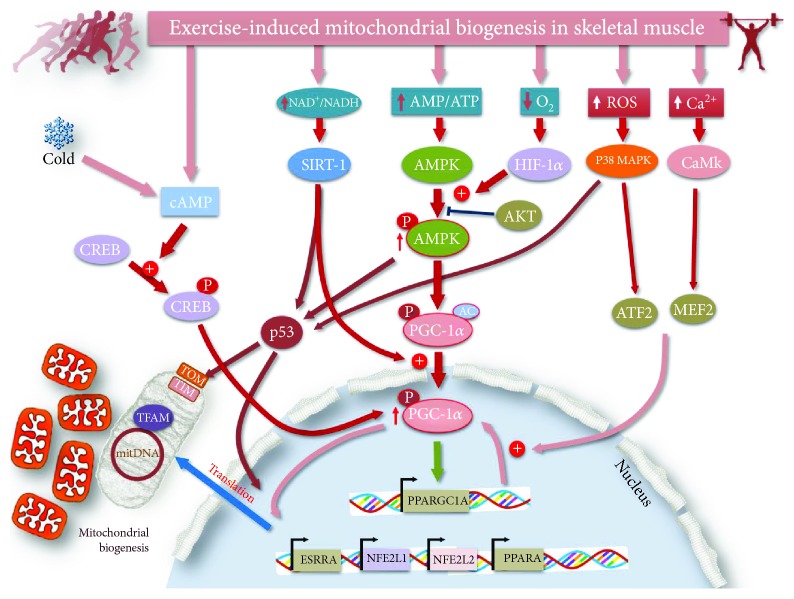
Exercise-induced mitochondrial signaling and biogenesis in skeletal muscle. ATP: adenosine triphosphate; ADP: adenosine monophosphate; NAD+/NADH: oxidized/reduced nicotinamide adenine dinucleotide; HIF-1*α*: hypoxia-inducible factors; p38 MAEK: P38 MAPK mitogen-activated protein kinase; CaMK: Ca^2+^/calmodulin-dependent protein kinase: AKT: serine/threonine-specific protein kinase; CREB: cAMP response element-binding protein; ATF2: activating transcription factor 2; MEF2: myocyte enhancer factor-2; AMPK: AMP-activated protein kinase; PGC-1: PPAR*γ* coactivator-1; PPAR*γ*: peroxisome proliferator-activated receptor *γ*; ROS: reactive oxygen species; SIRT-1: sirtuin 1; TFAM: mitochondrial transcription factor A; TIM: translocase of the inner mitochondrial membrane; TOM; translocase of the outer mitochondrial membrane; AC: acetylated; P: phosphorylated.

Within the mammalian skeletal muscle, mitochondria are mainly found forming large networks [48], which allows a rapid electrical interconnection between adjacent organelles [49]. This network highlights the plasticity of skeletal muscle mitochondria in response to cellular energetic challenges. Indeed, pioneering work by Palladin [50] showed that electrically stimulated skeletal muscle increased mitochondrial respiration. This work is also important because it introduced the concept of trainability with regards to mitochondrial function. In fact, in response to a single exercise session, there is a loss of homeostatic function that triggers a number of responses at the cellular (but also systemic) level to soften the next homeostatic challenge. For instance, if the homeostatic threat is triggered by endurance exercise (i.e., 60-90% VO_2_max) where the maintenance of ATP production mainly relies on the aerobic pathways, then the response will operate to improve such pathways. This effect is in accordance with the fact that endurance trained subjects show higher skeletal muscle mitochondrial volume than do sedentary subjects [51, 52]. The increase in mitochondrial volume seems to be one of the most important effects that improves endurance performance [53]. Indeed, in the present section, we will review the initial signals leading to an acute mitochondrial response (i.e., acute effect) and the repeated bout effect leading to a more permanent mitochondria adaptation (i.e., chronic effect). It should also be noted that mitochondria are highly plastic organelles that in response to the energetic challenges of the cell undergo several remodeling changes, which include [27] *de novo* mitochondrial biogenesis, mitochondrial fusion, and fragmentation of dysfunctional organelles (fission) for their breakdown by the autophagosome (mitophagy). It is important to note that all of these processes are triggered by acute and/or chronic exercise [54]. All these changes are detailed in the following paragraphs and depicted in Figure 4.

### 5.1. Early Exercise-Induced Signaling towards Mitochondrial Remodeling

Exercise is known to impact a number of intracellular signaling pathways that orchestrate mitochondrial responses and/or adaptations in skeletal muscle. It should be noted that these molecular pathways overlap each other, making it difficult to isolate one signal from another. Indeed, muscle contractile activity concomitantly triggers at least 4 intracellular signaling pathways that control mitochondrial function [55]: (1) increases in intracellular calcium mainly derived from the sarcoplasmic reticulum, (2) increases in ATP turnover leading to a rise in intracellular AMP, (3) increases in the ratio of nicotinamide adenine dinucleotide (NAD+) to its reduced form NADH (NAD+/NADH), and (4) increases in reactive oxygen species (ROS) production.

Experiments in cultured cells revealed that contraction-inducing calcium release regulates mitochondrial function by activating both calcium-calmodulin kinase (CaMK) and protein kinase C [56–58]. In addition, the rise in ATP demands within contracting muscle increases adenosine monophosphate (AMP) levels, leading to the activation of AMP-activated protein kinase (AMPK) [59]. AMPK is known to be a key regulator of metabolism through the inhibition of anabolism, which minimizes ATP consumption and stimulates catabolism to stimulate ATP production [60]. In fact, AMPK activity has been associated with increased mitochondrial content [61, 62] and function [63]. In addition to these kinases, exercise-induced ROS production increases the activity of mitogen-activated protein kinase 38 (p38 MAPK) [64]. Finally, the rise in NAD+ production directly activates the deacetylase SIRT1 [65] in a mechanism that may be potentiated by AMPK by directly increasing the NAD+/NADH ratio [66]. It should be highlighted that all of these signals directly or indirectly activate peroxisome proliferator-activated receptor gamma coactivator 1-alpha (PGC-1*α*) through phosphorylation, deacetylation, and/or regulation of its expression [55, 64, 67–69]. PGC-1*α* is a transcription factor that belongs to the peroxisome-activator receptor (PPAR) family. PGC-1*α*, together with other family members, such as PGC-related coactivator (PRC) and PGC-1*β*, regulates the expression of nuclear genes encoding mitochondrial proteins, thereby regulating mitochondrial content and function [70]. PGC-1*α* has been the best-studied family of nuclear receptors and accordingly is often referred to as the “master regulator” of mitochondrial biogenesis [55, 71, 72].

### 5.2. Mitochondrial Biogenesis

The definition of mitochondrial biogenesis is sometimes too general. In the present review, we will consider mitochondrial biogenesis as recently defined by Perry and Hawley [73]: “an expansion of total muscle mitochondrial volume.” This process requires a complex coordination of the transcription of the nuclear and the mitochondrial genome to assemble functional multisubunit respiratory chain proteins [52].

Early studies by John O. Holloszy [34] reported an association between mitochondrial content and exercise. More than thirty years later, the discovery of PGC-1*α* as a key controller of mitochondrial biogenesis shed more light on the molecular mechanisms triggered by exercise to increase mitochondrial content. As noted above, the activity of PGC-1*α* is coordinated by calcium, AMP, and ROS-related kinases, and all of these factors are highly sensitive to exercise-induced stress [74, 75]. These kinases regulate PGC-1*α* activity through phosphorylation. In addition, a single bout of exercise also increases PGC-1*α* activity through deacetylation by the NAD+-dependent deacetylase sirtuin 1 (SIRT1) [76]. However, the regulation of PGC-1*α* is not induced only at the posttranslational level. Indeed, it was observed that an acute bout of exercise increased the expression of PGC-1*α* [77–80]. Exercise-induced PGC-1*α* transcription is also controlled by CaMK and p38 MAPK, which phosphorylate the transcription factors myocyte enhancer factor 2 (MEF2) and activating transcription factor 2 (ATF2), leading to their translocation to the nucleus [81, 82]. Moreover, exercise induces PGC-1*α* translocation to the nucleus to both orchestrate mitochondrial biogenesis [83, 84] and self-regulate its own expression [85].

During the last decade, another transcription factor, the tumor suppressor protein p53, has also been identified as a powerful regulator of skeletal muscle mitochondrial content, function, and exercise capacity [86]. Research has found that p53 can be phosphorylated by p38 MAPK and AMPK [86–88]. Moreover, a single exercise session is known to deacetylate p53 by SIRT, which may help p53 to translocate to the nucleus [89]. Once in the nucleus, p53 regulates several nuclear-encoded mitochondrial proteins [90, 91]. Importantly, p53 can also be found inside the mitochondria in response to acute exercise, where it can affect mtDNA transcription [92]. Indeed, inside the mitochondria, p53 can form a complex with TFAM as well as with the mtDNA D-loop region, which controls the mitochondrial expression of some mitochondrial subunits. Therefore, p53 seems to be a transcription factor that can integrate the expression of both the nuclear and mitochondrial genomes in response to exercise. Mitochondrial biogenesis is often studied by using both biochemical and microscopy techniques. Exercise studies mainly use the activity, as well as the protein content, of citrate synthase (CS), succinate dehydrogenase (SDH), and cytochrome c oxidase (COX) as markers of mitochondrial biogenesis [55].

In addition, transmission electron microscopy (TEM) can be used to measure mitochondrial volume density (Mito_VD_) [45]. Moreover, mitochondrial respiratory function can be assessed from isolated mitochondria as changes in the mitochondrial ATP production rate and mass-specific mitochondrial respiration [93]. However, it has been observed in both rodents [94] and humans [93] that improvements in mitochondrial respiratory capacity can take place independently of changes in mitochondrial content. In an elegant study, healthy subjects were allocated for 4 weeks into 3 exercise groups: moderate continuous intensity training (MCIT), high-intensity interval training (HIIT), and sprint interval training (SIT) [95]. The results suggest that only SIT increases mitochondrial respiration, while the other groups showed no effect despite much higher training volumes. Another recent study showed that 6 weeks of MCIT showed an increase in Mito_VD_ without affecting mitochondrial respiration [45].

Therefore, it is likely that exercise intensity is a key aspect in regulating mitochondrial function. Interval exercise is commonly subdivided into HIIT and SIT. HIIT is often carried out at intensities >80% of the maximal heart rate and usually involves bouts of 1.5-16 min interspersed with 2-3 min of rest [96, 97]; SIT is usually performed as all-out exercise bouts lasting 6 to 90 s interspersed with periods of rest of 1 to >5 times the duration of the exercise [98]. MCIT is performed in a continuous manner and at lower intensities than internal exercise is. In recent years, a number of studies have investigated the mitochondrial adaptations induced by different exercise regimens. A recent paper by Perry et al. [99] analyzed PGC-1*α* mRNA expression during 7 HIIT sessions carried out over 2 weeks. Their data may suggest that intense exercise induces stronger and more rapid mitochondrial adaptation than MCIT does. However, it has been reported that SIT [100] and MCIT [74] increase CS activity to a similar extent 24 h following an acute exercise bout. Instead, exercise intensity may be an efficient approach to elicit mitochondrial adaptations. Indeed, two studies are aimed at comparing the mitochondrial adaptations elicited by two weeks of either SIT or MCIT [101, 102]. They found similar increases in both CS and COX activities even though MCIT comprised 4.5 h of weekly training, while SIT comprised only 10 min of effective weekly training. However, it remains unclear how extremely low-volume SIT induces such an efficient effect. It has been suggested that SIT-induced ROS production might be a possibility [103]. Accordingly, it has been observed that SIT-induced ROS production over two weeks of training induces the fragmentation of the ryanodine receptor (RyR), leading to postexercise Ca^+^ accumulation [104]. This finding is in accordance with the observation that calcium-related signaling seems to be important in response to acute SIT exercise [105]. In addition, a more recent study highlighted that *β*-adrenergic stimulation, muscle lactate accumulation, and skeletal muscle pH reduction seem to be important to elicit the SIT-related molecular cascade leading to mitochondrial biogenesis [75]. Therefore, the above studies suggest that mitochondria respond rapidly to exercise stimuli and that intense exercise of short duration may be an efficient strategy to achieve mitochondrial adaptations.

In addition, it seems plausible that different exercise intensities elicit divergent mitochondrial adaptations. In a recent study, subjects performed six sessions of HIIT with one leg, while the other leg performed MCIT, with similar volumes between legs [106]. In these subjects, HIIT was the only intervention that increased CS maximal activity and OXPHOS capacity. It should, however, be noted that neither HIIT nor MCIT increased mitochondrial respiration as assessed by OXPHOS capacity normalized to CS maximal activity [103]. Indeed, another study compared the effects of 4 weeks of MCIT-, HIIT-, and SIT-based training in healthy subjects [95]. The authors found that although all the interventions increased the expression of the transcription factors p53 and PGC-1*α*, only SIT increased mass-specific mitochondrial respiration. It can be argued that the higher the intensity, the more type 2 fibers are recruited [35]; thus, the increased mitochondrial respiration could be due to phenotype changes. However, the best controlled study (regarding its within-subject design) showed no differences between MCIT and HIIT in COX activity within type 2 skeletal muscle fibers [103]. In contrast, in a recent review, the authors provide valuable relationships between mitochondrial adaptations and exercise intensity by plotting the data of available human studies [93]. Among the truly important observations, it should be highlighted that the authors conclude that high-volume low-intensity (i.e., MCIT) training is important to increase mitochondrial content. However, exercise intensity seems to be important in increasing mass-specific mitochondrial respiration [93]. Therefore, divergent exercise stimuli may trigger different molecular pathways leading to different mitochondrial properties and possibly dynamics.

### 5.3. Mitochondrial Dynamics

**Figure 5:**
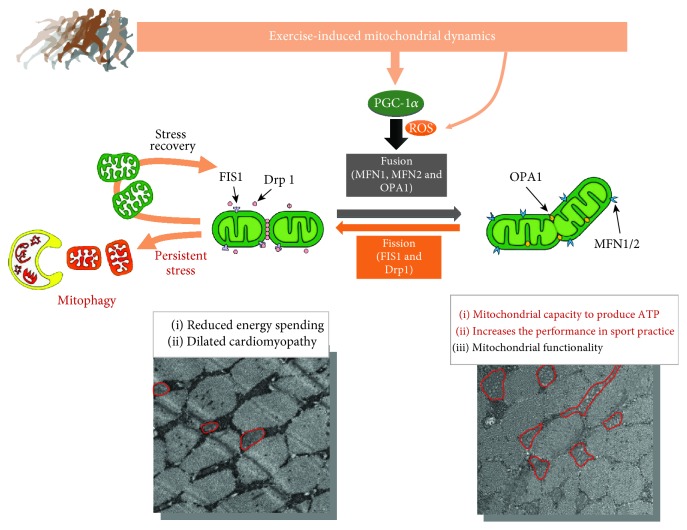
Mitochondrial dynamics is affected by/during exercise. PGC-1: PPAR*γ* coactivator-1; ROS: reactive oxygen species; FIS1: mitochondrial fission 1 protein; Drp1: dynamin-related protein 1; OPA1: mitochondrial dynamin like GTPase; MFN1/2: mitofusin 1 and 2 protein.

Mitochondria can undergo dynamic morphological changes that involve crista remodeling, fusion, and fission, which collectively regulate cellular function [27]. Indeed, mitochondria not only regulate their shape to adapt to the energetic needs of the cells but also maintain an internal quality control in which damaged mitochondria are eliminated by the autophagosome. During exercise, the higher energetic demands acutely induce mitochondrial morphological changes, which may indeed regulate long-term mitochondrial bioenergetics [54] and help to maintain mitochondrial functions (Figure 5).

In rodents, it was recently shown that acute running exercise increases dynamin-related protein 1- (DRP1-) induced mitochondrial fission and mitophagy efflux [107]. In addition, using a combination of transmission electron microscopy and molecular biology, Lavorato et al. [108] reported that both exercise running to exhaustion and electrical stimulation result in mitochondrial fission in a DRP1-dependent manner. Therefore, in rodents, acute exercise seems to activate DRP1 pathways, leading to mitochondrial fission and increased mitophagy efflux; however, the mechanisms are yet to be elucidated. It was initially thought that exercise-induced AMPK phosphorylation at Thr172 was the upstream mechanism triggering mitochondrial fragmentation and mitophagy [54]. However, more recent studies suggest that AMPK is responsible for exercise-induced mitophagy but not DRP1-related fission [107]. In this regard, mitochondrial fission factor (MFF) is a mitochondrial outer membrane that involves the recruitment of DRP1 from the cytosol to the mitochondrial surface to catalyze the fission reaction. Notably, it has been demonstrated that mitochondrial stress induced MFF phosphorylation by AMPK, thus increasing mitochondrial fission [109]. Thus, AMPK seems to couple fission and mitophagy in response to cellular stress. However, whether exercise-induced AMPK activation results in MFF-related fission has yet to be determined. Interestingly, during atrophy induced by denervation or fasting, there is massive mitochondrial fission, leading to an exacerbated autophagy that leads to muscle wasting. This event can be prevented by expressing a Drp1-negative mutant and knocking down AMPK [110], suggesting that the Drp1-AMPK axis is involved not only in exercise-induced autophagy but also in muscle atrophy-induced autophagy. While the first seems to have a role in quality control, the latter is an extreme event resulting in deleterious effects. Whether it is only a quantitative matter or whether the activation of these molecular mechanisms requires different interactors is still to be discovered. A very recent paper reported that Drp1-/- mice have altered muscle adaptation upon exercise that results in reduced running performance. This finding definitively demonstrates that fission and Drp1 in particular are key elements for muscle activity and physiology [111].

On the other hand, mitochondrial fusion and elongation of the mitochondrial network have been proposed to occur in response to cellular energetic reductions [26, 112] and are regulated by three GTPases: mitofusin 1 (MFN1), mitofusin 2 (MFN2), and optic atrophy 1 (OPA1). Once two mitochondria come into close contact, MFN1 and MFN2 change their molecular conformation to permit mitochondrial outer membrane fusion [113]. Inner membrane mitochondrial fusion is known to be regulated by OPA1 [27]. In addition, MFN expression seems to be regulated by PGC-1s and by the estrogen-related receptor (ERR) [114, 115]. In fact, exercise-induced PGC-1*α* translocation to the skeletal muscle nucleus is known to induce MFN1 and MFN2 expression in human skeletal muscle in an ERR-dependent manner [116]. However, direct evidence of exercise-induced mitochondrial fusion is lacking. Picard et al. [117] showed that acute voluntary running in mice increases electrodense mitochondrial contact sites, but no effect on MFN2 and OPA1 was observed. In addition, a recent study using skeletal muscle PGC-1*α* KO mice showed that exercise-induced mitochondrial network elongation is dependent upon PGC-1*α* suppression of mitochondrial fission 1 (FIS1) rather than MFNs or OPA1 [118]. Therefore, within exercised skeletal muscle, PGC-1*α* may promote a profusion environment by suppressing fission activity.

In humans, it has been observed that long-term exercise induces an enlargement of the mitochondrial network [45, 46], which may help to electrically interconnect the sarcolemma and the core of the muscle cell, thereby linking oxygen rich areas to those areas with limited oxygen availability [44, 49]. As mitochondrial fusion occurs in parallel with skeletal muscle energetic reduction [112], it seems plausible that acute exercise may also induce rapid mitochondrial fusion events. However, studies aiming to study the effects of either acute or long-term responses to exercise on mitochondrial fusion are lacking. Cartoni et al. [116] reported that well-trained cyclists performing strenuous continuous cycling showed increases in MFN mRNA levels but not protein levels in the 24 h following the exercise.

More recently, it has been reported that both SIT and MCIT lead to rapid MFN2 mRNA expression [84]. In addition, Fiorenza et al. [75] showed that both MCIT and SIT but not extremely low-volume SIT increased MFN2 mRNA levels 3 h postexercise. Regarding the cumulative effect of several weeks of training, it has been reported that in previously untrained subjects, MFN2 protein content increases following 6 weeks of MCIT [45]. In another study, moderately trained individuals were allocated to SIT, HIT, and MCIT for four weeks [95]. The results show that all of the exercise interventions increased skeletal muscle MFN2 protein content. In addition, in an elegant study, Perry et al. [99] studied time-course responses of skeletal muscle signaling in moderately trained subjects during two weeks of HIIT. They found that MFN1 but not MFN2 protein content increases at the end of the training period.

These results show the complexity of the mitochondrial fusion machinery. In addition, discrepancies between studies could be due to differences in the subject's characteristics and/or methodological issues. Interestingly, OPA1, the master of mitochondrial cristae, has been found to be downregulated in biopsies of older sedentary humans but not older sportsmen, suggesting that exercise counteracts aging-related sarcopenia [119]. To understand the molecular mechanism, the authors specifically depleted Opa1 in the muscle, and they demonstrated that the lack of OPA1 induces mitochondrial dysfunction, leading not only to an impairment of muscle activity but also to precocious senescence and degeneration of multiple organs caused by the activation of ER stress and the unfolded protein response (UPR) mediated by an increase in serum levels of FGF21. This paper [119] not only identified OPA1 as a sensor of exercise but also demonstrated for the first time how the modification of mitochondrial activity through morphology can have a systemic effect, underlining the importance of a holistic approach to study mitochondria.

Moreover, the molecular mechanisms underlying mitochondrial fusion in response to exercise remain to be elucidated, and further studies combining molecular signaling and microscopy technology can help to unravel mitochondrial dynamics in response to exercise.

In addition, other factors can also affect mitochondrial dynamics in response to exercise. For instance, it has been reported that as early as the third HIT session, both FIS1 and DRP1 protein contents increase in moderately trained subjects [99]. However, this report is in contrast with recent results showing a stronger profusion rather than profission environment within the skeletal muscle of aged subjects subjected to six weeks of HIIT [120]. This discrepancy could occur because aged skeletal muscles show a blunted mitophagy capacity, leading to the accumulation of damaged mitochondria [121]. In addition, the phenotype of the skeletal muscle studied may also impact the mitochondrial responses to exercise. In fact, it has been reported that two weeks of SIT engaging both the arms and legs or the legs but not the arms increased mitochondrial respiration [122]. This effect likely occurs because the arms and legs show different phenotypes [123, 124]. Therefore, mitochondrial dynamics in response to exercise seems to be an interesting area for future research. Future studies should determine the molecular mechanisms underlying mitochondrial fission and fusion in response to exercise in health and disease. Moreover, skeletal muscle phenotypic differences regarding mitochondrial dynamics in response to exercise may help to shed light on the effects of exercise on mitochondrial and cellular metabolism.

### 5.4. Mitochondrial Function and SCs

Regular exercise increases mitochondrial crista density, thereby improving cellular function and ultimately whole-body VO_2_max [124]. Since cristae are the privileged site of the OXPHOS system [125] whose shape is fundamental for the assembly and stability of SCs [21], we can hypothesize that increasing crista density could increase SC formation. Recent studies have observed that endurance exercise induces mitochondrial complex assembly into SCs. In rats, ten weeks of treadmill running increases SC assembly, thereby improving mitochondrial oxidative status [126]. In a very elegant study, Amati's group [127] demonstrated how 4 months of moderate-intense aerobic exercise increases muscle respiration in older sedentary adults. From the molecular point of view, they found that all respiratory complexes, but particularly CI, were upregulated and redistributed to SCs. The overall result is that SC formation is enhanced upon exercise and correlates with better mitochondrial respiration. This work reveals for the first time in humans how SC formation could be an adaptive response to increase mitochondrial respiration and satisfy higher energetic demand [127]. Moreover, it has been proposed that SIT but not MCIT increases mitochondrial respiration without increasing mitochondrial volume. This observation suggests that exercise intensity might be an important determinant in SC assembly.

Although SIT protocols are known to increase circulating lactate levels over 13 mmol/L [128, 129], the repeated sprint bout effect on oxidative phosphorylation described by Parolin et al. [29] may suggest an impact on mitochondrial SC assembly. Related to the advantages of SC formation already discussed in a previous section (Mitochondria: The Hub of Energy Production), the real advantage of having a higher SC/complex ratio is still under investigation, but it is now clear that situations involving impaired mitochondrial metabolism are correlated with less SC formation, as observed during aging [130], heart failure [131], and diabetes [132]. An additional advantage of SCs could be enhancing the enzymatic activity of the complexes, thus improving overall mitochondrial respiration. An early study measured CI and CIV activity from I+III_2_ and I+III_2_+IV isolated from bovine heart by Blue Native PAGE in-gel activity assay and by spectrophotometric activity after electroelution assay of the SC structures [133]. The results indicate more activity in the supercomplex-assembled CI and CIV. Later, different works confirmed this observation, especially in nervous system cells from animal models of Parkinson's disease [134–136], and Amati's work confirmed this finding in muscle [127]. However, even though these results seem to support this hypothesis, we must consider important technical details. Indeed, all of these results have been obtained either by Blue Native in-gel activity assay or spectrophotometric activity assay, which both have technical limitations. The former is a nonlinear nonquantitative assay, while the latter measures the total amounts of complexes and cannot discriminate the specific activity of assembled vs. not assembled complexes. Considering these aspects, we believe that the *in vitro* results are valid approximations but definitive experimental confirmation *in vivo* is still needed. Another fundamental question, which is still open, is which molecular mechanisms trigger exercise-induced SCs assembly.

It has been reported that the lipid composition of the inner mitochondrial membrane is important for SC stabilization3, but it has never been addressed whether lipid composition may change upon exercise. As explained previously, OPA1 is a sensor of exercise that is downregulated in old sedentary individuals [119]. Moreover, considering its importance for SC maintenance [21], we could speculate that increasing OPA1 content might be a molecular mechanism to increase SC formation upon exercise. Another critical element for the assembly of SCs is SCAF1 [12], which could be a molecular target of exercise to increase SC formation, but there is still no investigation in this direction. Those elements could be all targets of exercise-dependent SC formation, but further investigation is needed to understand how exercise impacts the assembly of SCs and their role in adaptive processes upon increasing energy demand.

## 6. Use of Mitochondrial Active Nutraceuticals to Improve Exercise

The present review highlights the wide range of adaptations that exercise training can elicit within skeletal muscle mitochondria. In addition, several studies have attempted to stimulate such effects by stimulating particular pathways using so-called exercise-mimetic compounds. In fact, Narkar et al. [137] proposed that pharmacological activation of AMPK activity in mice is enough to change the skeletal muscle phenotype towards a more oxidative phenotype. This possibility attracted the attention of exercise physiologists aiming at discovering new nutritional supplements to improve skeletal muscle performance and health.

Polyphenols are naturally occurring compounds with similar structures (several hydroxyl groups on aromatic rings) and are mainly found in fruits and vegetables. Polyphenols are known to regulate free radical scavenging, cellular signaling and transduction, gene expression, and cellular communication [138]. Two of the best-studied polyphenols are resveratrol, which is found in red wine, and quercetin, which is mainly found in apples and onions [139]. Both quercetin and resveratrol have been extensively studied by exercise physiologists, as they are known to promote mitochondrial biogenesis through PGC-1*α* signaling by targeting SIRT1 [140–143]. Accordingly, mice supplemented with either resveratrol or quercetin are known to increase their endurance running performance [142, 144]. Therefore, polyphenol supplementation may change the skeletal muscle phenotype and thereby improve overall physical fitness.

However, the results of human studies aiming at finding an ergogenic or healthy effect of polyphenols are contradictory. For instance, it has been reported that 30 days of resveratrol supplementation in obese subjects improves cardiovascular function and skeletal muscle metabolism [145]. However, in healthy postmenopausal women, there were no effects on cardiovascular outcomes, lipid metabolism, or glucose metabolism after 12 weeks of resveratrol supplementation [146]. Moreover, Glieman et al. [147] showed that resveratrol supplementation during exercise blunted the cardiovascular adaptations induced by exercise in older subjects. Notably, young subjects also show a blunted effect of resveratrol on the skeletal muscle adaptations induced by exercise [148]. Therefore, the health-related effects of resveratrol could be more evident in diseased than in healthy subjects, but there is also a negative interaction induced by resveratrol on exercise adaptations.

In agreement with this notion, quercetin is known to increase skeletal muscle SIRT1 mRNA levels in sedentary but not trained animals [143]. However, human studies also show contradictory data. It has been reported that supplementation with 1000 mg/d quercetin for 2 weeks increases intense cycling performance in sedentary subjects [149]. Notably, no effect of quercetin on ventilation, heart rate, or oxygen consumption was observed during 60 min of low-intensity steady-state cycling. In addition, MacRae and Mefferd [150] supplemented a cocktail of antioxidants with or without 300 mg of quercetin for 6 weeks in well-trained subjects. They found that those consuming quercetin increased exercise performance during a 30 km cycling time-trial. It should, however, be noted that all the cocktails contained 45 mg of caffeine, which could, at least, interfere with the observed results. In addition, Davis et al. [151] observed increases in VO_2_max and ride time to exhaustion in untrained subjects following 7 days of consumption of 1000 mg/d quercetin. However, a meta-analysis classified the effects of quercetin on exercise performance as unlikely to translate into a real ergogenic effect [152]. In fact, Cureton et al. [153] designed a study in which several endurance and time-trial tests were analyzed following at least 7 days of supplementation with 1000 mg/kg quercetin. They found no ergogenic effect of quercetin; moreover, they found that skeletal muscle PCr recovery following 1 min of isometric contraction was unaffected by quercetin. In addition, well-trained subjects consumed chewing gum containing 1000 mg of quercetin and other antioxidants, while subjects in the control group consumed the same chewing gum without quercetin [149]. The subjects consumed these chewing gums for 14 days, during 3 days of intensified training period and for an additional 7-day period. The investigators found that quercetin improved the immunological response to the intensified training period but failed to detect changes in exercise performance and skeletal muscle mitochondrial biogenesis markers. Collectively, these findings suggest that in contrast to mouse studies, quercetin does not improve mitochondrial function in either sedentary or trained humans. In addition, any performance-related effect, which is more likely in sedentary subjects, may be due to an enhanced immunological response to exercise.

It is noteworthy that in either mice [143] or humans [147, 148], polyphenol supplementation seems to interfere with skeletal muscle adaptations to exercise. This harmful effect has not been studied in detail; however, the antioxidant properties of polyphenols may be the underlying mechanism. Indeed, an acute bout of exercise induces ROS production, and this ROS release is attenuated as the skeletal muscle adapts to exercise [154]. This observation suggests that exercise training induces so-called oxidative eustress, which is important for health because it maintains a balance between oxidants and antioxidants [155]. Accordingly, exercise-induced cellular adaptations are triggered by the signals transduced by the ROS produced at the mitochondrial and extramitochondrial levels [156, 157]. Accordingly, vitamin C and E supplementation during exercise impedes exercise-induced skeletal muscle adaptations in both humans and rats [158, 159]. It will therefore be of great value to identify whether different doses of polyphenols combined with exercise may target key mitochondrial pathways without affecting the redox balance elicited by exercise.

In addition, much attention has been paid to dietary nitrate (NO_3_^−^) supplementation, as it has been linked to improvements in human exercise performance in a wide range of physical activities [160]. Among these effects, it is important to highlight that nitrate supplementation can reduce the O_2_ cost of submaximal exercise. In fact, three to six days of nitrate supplementation enhances the mitochondrial coupling efficiency, thereby improving skeletal muscle P/O [161, 162]. However, more research is needed in this area because similar to the other supplements discussed above, the ergogenic potential of nitrate is blunted when combined with high-intensity exercise [163].

Finally, we would like to consider the gut microbiota, as it shares the property of nutrient metabolism with mitochondria [164]. In addition, it has been observed that the host's fitness is related to gut immune function and microbiota function [165, 166]. Moreover, a bidirectional cross-talk between the gut microbiota and mitochondria has been proposed. In fact, this symbiotic-like relationship between the gut microbiota and mitochondria seems to be favored by exercise training [167]. Thus, the regulation of the mitochondria-gut axis in response to exercise will be a fertile area of research for years to come.

## 7. Therapeutic Use of Exercise

Exercise evokes a multisystem response that leads to health outcomes. In fact, exercise can be a useful tool for the treatment of a number of metabolic diseases [168, 169]. Exercise can revert the deleterious effects of disuse and aging [170] and has positive effects on type 2 diabetes and cardiovascular disease [171]. Indeed, it is currently widely accepted that exercise is medicine. However, this concept requires elucidation of the limit between physiological exercise and deleterious exercise to find a balance in the personalization of exercise [172]. The World Health Organization (WHO) and American Heart Association (AHA) recommend 150 minutes of moderate intensity physical activity or 75 minutes of vigorous physical activity weekly. The time intervals should be at least 10 minutes or more per session. However, these recommendations are currently under review, as it is unclear which exercise doses (i.e., time × intensity) are better to maximize the health-related effects of exercise. For instance, it has been recently reported that cardiorespiratory fitness is associated with reduced long-term mortality, and no upper limit of this benefit was observed [173]. This finding is in line with recent advice to perform three to five times the WHO physical activity recommendations to achieve maximal benefits [168].

In addition to the exercise dose, it is also important to highlight that the time of the day at which exercise is performed may also limit health benefits in subjects suffering from type 2 diabetes [174] and hypertension [175]. Therefore, future research must characterize the best exercise doses and regimens for both diseased and healthy populations. In addition, the implications of circadian rhythms for exercise-induced health benefits will also be an important field of research in upcoming years. However, it is clear that regular moderate intensity exercise is an easy and cost-free intervention to improve overall health.

Regarding the main topic of the present review, it should be noted that mitochondrial dysfunction is associated with aging, type 2 diabetes, and cardiovascular disease [176–178]. Although it is currently unclear whether mitochondrial dysfunction is a cause or a consequence of the disease, recent studies in rodents suggest that mitochondrial dysfunction triggers muscle atrophy in response to metabolic disease [179]. Therefore, exercise may induce, at least in part, its health benefits by improving mitochondrial function. In addition, reduced respiratory SC assembly has been observed in type 2 diabetic subjects [132] as well as in the aged heart [130] and the brain cortex of aged rats [180]. In addition, mitochondrial crista disruption is an important process that leads to heart failure [181]. These observations highlight the importance of maintaining mitochondrial function in general and the mitochondrial SCs in particular to prevent some metabolic diseases. Studies on how different types and doses of exercise affect SC organization in these diseases may help to shed light on the key molecular pathways involved in these metabolic diseases.

## 8. Future Perspectives

The effects of different exercise regimens on mitochondrial function need to be further studied. Of particular relevance seems to be the effect of exercise on mitochondrial fusion and fission in aged and diseased populations. In addition, whether exercise can improve mitochondrial P/O and skeletal muscle contraction efficiency due to mitochondrial SCs assembly is another important topic in the field.
