# Corrigendum to “Stay Fit, Stay Young: Mitochondria in Movement: The Role of Exercise in the New Mitochondrial Paradigm”

**DOI:** 10.1155/2021/9274841

**Published:** 2021-01-18

**Authors:** Jesus R. Huertas, Rafael A. Casuso, Pablo Hernansanz Agustín, Sara Cogliati

**Affiliations:** ^1^Institute of Nutrition and Food Technology, Biomedical Research Centre, Department of Physiology, University of Granada, Granada, Spain; ^2^Centro Nacional de Investigaciones Cardiovasculares (CNIC), Madrid, Spain

In the article titled “Stay Fit, Stay Young: Mitochondria in Movement: The Role of Exercise in the New Mitochondrial Paradigm” [1], the authors would like to make a clarification by adding the following sentence to the abstract:

“The duration of maximal exercise at which equal contributions are derived from the anaerobic and aerobic energy systems appears to occur between 1 and 2 minutes and most probably around 75 seconds, a time that is considerably earlier than has traditionally been suggested.”
